# The Human TUT1 Nucleotidyl Transferase as a Global Regulator of microRNA Abundance

**DOI:** 10.1371/journal.pone.0069630

**Published:** 2013-07-18

**Authors:** Emily C. Knouf, Stacia K. Wyman, Muneesh Tewari

**Affiliations:** 1 Human Biology Division, Fred Hutchinson Cancer Research Center, Seattle, Washington, United States of America; 2 Molecular and Cellular Biology Graduate Program, University of Washington, Seattle, Washington, United States of America; 3 Public Health Sciences Division, Fred Hutchinson Cancer Research Center, Seattle, Washington, United States of America; 4 Clinical Research Division, Fred Hutchinson Cancer Research Center, Seattle, Washington, United States of America; 5 Department of Medicine, University of Washington, Seattle, Washington, United States of America; French National Center for Scientific Research - Institut de biologie moléculaire et cellulaire, France

## Abstract

Post-transcriptional modifications of miRNAs with 3′ non-templated nucleotide additions (NTA) are a common phenomenon, and for a handful of miRNAs the additions have been demonstrated to modulate miRNA stability. However, it is unknown for the vast majority of miRNAs whether nucleotide additions are associated with changes in miRNA expression levels. We previously showed that miRNA 3′ additions are regulated by multiple nucleotidyl transferase enzymes. Here we examine the changes in abundance of miRNAs that exhibit altered 3′ NTA following the suppression of a panel of nucleotidyl transferases in cancer cell lines. Among the miRNAs examined, those with increased 3′ additions showed a significant decrease in abundance. More specifically, miRNAs that gained a 3′ uridine were associated with the greatest decrease in expression, consistent with a model in which 3′ uridylation influences miRNA stability. We also observed that suppression of one nucleotidyl transferase, TUT1, resulted in a global decrease in miRNA levels of approximately 40% as measured by qRT-PCR-based miRNA profiling. The mechanism of this global miRNA suppression appears to be indirect, as it occurred irrespective of changes in 3′ nucleotide addition. Also, expression of miRNA primary transcripts did not decrease following TUT1 knockdown, indicating that the mechanism is post-transcriptional. In conclusion, our results suggest that TUT1 affects miRNAs through both a direct effect on 3′ nucleotide additions to specific miRNAs and a separate, indirect effect on miRNA abundance more globally.

## Introduction

Because microRNAs (miRNAs) are critical regulators of key processes such as development and differentiation, their expression levels must be precisely regulated. Recent progress has been made in understanding regulation of miRNA transcription and multiple stages of biogenesis (i.e., processing) [1], [2]. However, the regulation of miRNA degradation is poorly understood in humans. Most studies describe miRNAs as being remarkably stable, with reported half-lives for human miRNAs ranging from hours to several days [3], [4]. In plants and worms, exonucleases that degrade miRNAs have been identified [5], [6], but in humans no global mechanisms for regulating the turnover of miRNAs have been described.

We and others have observed that mature miRNAs show a diversity of nucleotide additions at their 3′ end in metazoan miRNA high-throughput sequencing studies [7]–[12]. These 3′ additions have recently been demonstrated to be a conserved, biological phenomenon in humans, mice, plants, and flies [13]–[22]. The origins of miRNA 3′ additions can be diverse, and include both aberrant enzymatic processing as well as post-transcriptional modification of precursor and mature miRNAs [23]–[26]. MicroRNA variants, commonly referred to as “isomiRs”, substantially expand the known miRNA transcriptome; however, the biological significance of 3′ additions is not yet well-understood. In plants, methylation and adenylation rendered some miRNAs resistant to degradation [27], [28]. In contrast, uridylation by nucleotidyl transferase enzymes led to increased degradation of specific miRNAs in both plants and algae [29]–[31]. Taken together, these studies demonstrate that the modification of a miRNA with a single nucleotide addition can be sufficient for altering miRNA decay.

In humans, the role of 3′ additions is poorly understood, as only a handful of reports have examined the effects of A and U additions on the stability of a small number of miRNAs. The addition of a 3′ A to the miRNA miR-122 was associated with increased abundance of the miRNA [32]. On the other hand, the addition of a 3′ U to miR-26a did not change the abundance of the miRNA, but instead prevented the miRNA from repressing its mRNA target [33]. Recent work has suggested that 3′ additions may be involved in miRNA turnover, as increased uridylation of an exogenously expressed miRNA has been observed during its decay [34]. MicroRNA isomiRs are found in association with the Ago proteins, although nucleotide additions were shown to reduce miRNA association with certain Ago proteins in humans [16]. Therefore, 3′ additions have been hypothesized to have miRNA-specific effects on controlling either the targeting or stability of a miRNA.

In humans, many miRNAs feature 3′ non-templated additions of an adenosine (A) or uridine (U), which do not match the genomic or precursor sequences [25], [26], [35], and thus are not ambiguous in origin. Non-templated 3′ additions are regulated by multiple members of the nucleotidyl transferase family in humans. We and others have found that the PAPD4 enzyme governs the 3′ A additions of a broad panel of miRNAs [25], [26]. We also identified multiple other enzymes, including TUT1, PAPD5, MTPAP, ZCCHC11, and ZCCHC6, that regulate 3′ additions in a miRNA-specific manner [25]. In order to investigate the biological significance of miRNA 3′ additions, here we examined the relationship between changes in miRNA additions and miRNA abundance following suppression of each of these nucleotidyl transferases. We found that among the miRNAs we examined, those with increased 3′ addition–particularly of a 3′ U–were associated with decreased miRNA abundance. We also identified a nucleotidyl transferase, TUT1, which exerts broad effects on miRNA expression levels despite its seemingly restrictive regulation of 3′ additions to specific miRNAs. This represents a new role for the TUT1 nucleotidyl transferase, which has previously been shown to regulate the stability of a subset of mRNA transcripts and the U6 snRNA [36]–[38]. Taken together, our studies demonstrate that TUT1 serves as not only an important modulator of 3′ additions to specific miRNAs, but also more globally as a post-transcriptional regulator of the abundance of a large number of miRNAs through an indirect mechanism.

## Results and Discussion

### Suppression of Specific Nucleotidyl Transferases Leads to Increases as well as Decreases in miRNA 3′ Nucleotide Additions

**Figure 1:**
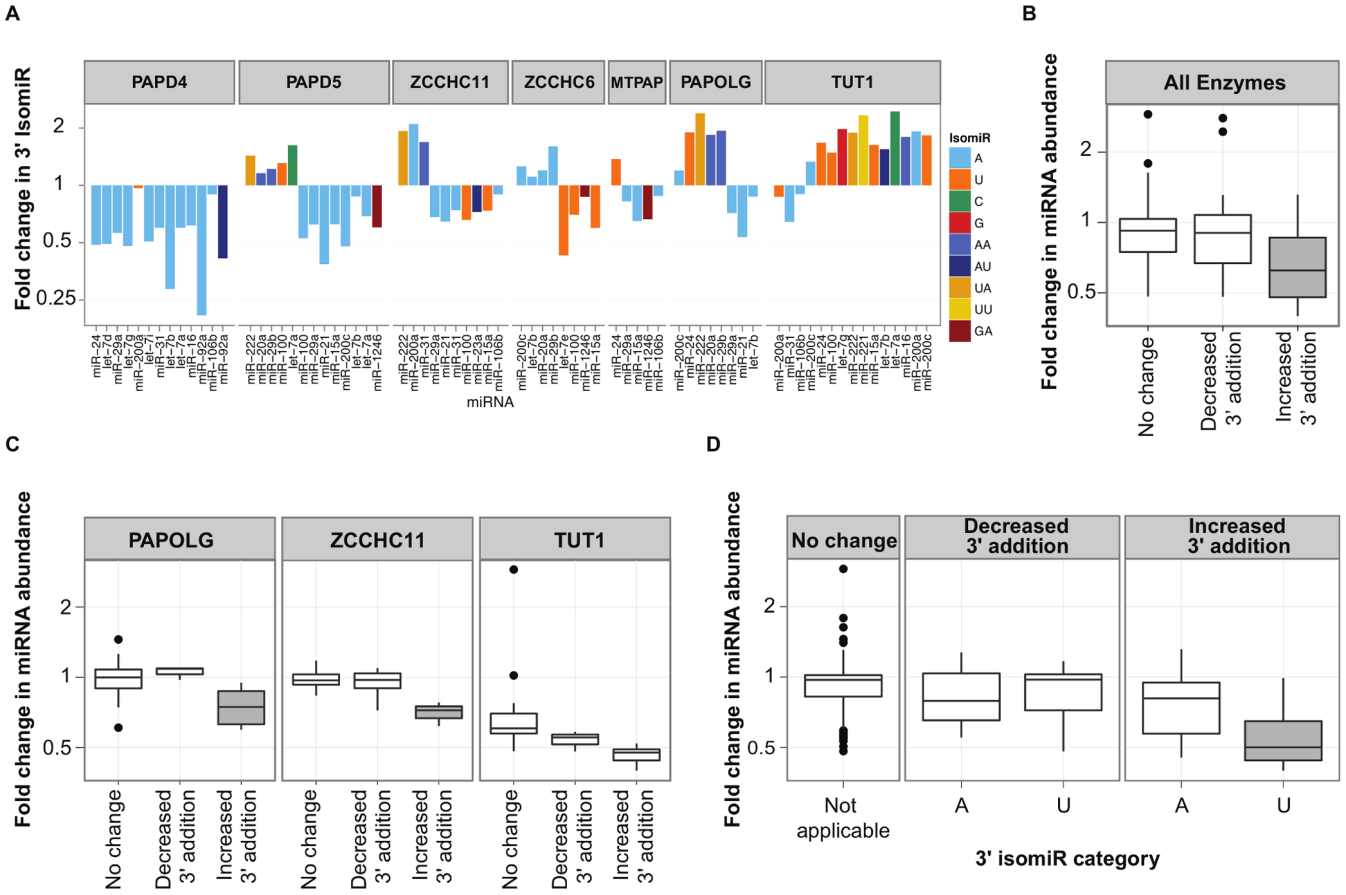
Association between changes in miRNA 3′ nucleotide additions and changes in miRNA abundance. MicroRNA 3′ additions and total abundance levels were assessed with NanoString profiling following the suppression of nucleotidyl transferases in HCT-116 cells. (A) Bars depict miRNAs with significant changes in additions compared to negative control cells with a false discovery rate <10%. The bars display the fold change in a given miRNA isomiR, with the color of the bar indicating the 3′ addition affected. The enzyme names above the bars indicate the suppressed nucleotidyl transferase. Data for miRNAs showing decreased addition are from our previous report [25]. (**B**) Boxplots comparing the abundance changes of miRNAs showing increases, decreases, or no change in 3′ additions. MicroRNAs with significant increases in 3′ additions feature a statistically significant decrease in abundance compared to miRNAs with unchanged or decreased additions (t-test, p-value <0.005). (**C**) Increased additions are associated with decreased miRNA abundance following suppression of three different nucleotidyl transferase enzymes. Shading of a box indicates a t-test p-value <0.05 when comparing the abundance in miRNAs with unchanged versus increased or decreased 3′ addition. (**D**) MicroRNAs with increased 3′ U additions show significant decreases in abundance compared to miRNAs with unchanged additions (t-test, p-value <2×10^−5^).

To investigate the association between miRNA 3′ additions and miRNA abundance, we developed a system to perturb 3′ nucleotide additions to endogenous miRNAs. We have shown that multiple nucleotidyl transferase enzymes regulate miRNA 3′ additions in an enzyme and miRNA specific manner [25]. To assay miRNA 3′ additions and abundance in a quantitative manner, we employed the nCounter miRNA platform from NanoString Technologies [39], which we adapted to assess the expression of approximately 130 canonical miRNAs and their two most common 3′ variants [25]. In our previous study, we described specific miRNA 3′ additions that were reduced in frequency following the individual suppression of seven different nucleotidyl transferase enzymes in HCT-116 colon cancer cells, with most enzymes found to be mediating 3′ A additions. However, in a more extended analysis of the data, we have also found that certain miRNA 3′ additions increase in response to enzyme suppression (**Figure 1A**). For example, knockdown of the enzyme TUT1 led to significant increases in additions in 11 out of the 24 miRNAs that had isomiRs robustly detected by the NanoString platform (<10% FDR, Benjamini-Hochberg method). The diversity of additions added to the miRNAs was high. In contrast to the preponderance of 3′ A additions that were lost following enzyme suppression, we saw significant increases in 3′ U, UA, UU, and other additions (<10% FDR, Benjamini-Hochberg method, **Figure 1A**). These results indicate that nucleotidyl transferases have variable effects on miRNAs, with some members of the family primarily promoting additions (e.g., PAPD4) whereas other members can either promote or potentially prevent 3′ additions, depending upon the specific miRNA and type of addition involved. We evaluated whether any nucleotidyl transferases showed compensatory increases in expression following the suppression of TUT1, the enzyme whose loss led to the greatest number of increased miRNAs additions. We found that TUT1 reduction led to minimal changes in the mRNA expression of other nucleotidyl transferases (**Figure S1A**), although effective antibodies to determine the protein expression of the entire panel of nucleotidyl transferases were not available. Therefore, while the mechanism of compensatory 3′ additions remains unknown, our results indicate that increased 3′ additions are a frequent occurrence when the activity of specific nucleotidyl transferases is disrupted. Importantly, our suppression of nucleotidyl transferase enzymes also created a diversity of altered 3′ additions: Some 3′ additions were gained and others were lost, which enabled us to examine the association between each altered addition and total miRNA abundance.

### Increased 3′ U Addition is Associated with a Significant Decrease in miRNA Abundance

The relationship between 3′ additions and miRNA abundance has not been described on a global scale in humans. Because 3′ additions can modulate miRNA stability in other organisms, we hypothesized that changes in additions would affect the total abundance levels of miRNAs in humans. We examined the expression levels of miRNAs that displayed significant increases and decreases in 3′ additions following our suppression of nucleotidyl transferases. To quantitatively compare abundance levels, we used the NanoString nCounter platform, as this system does not involve sample amplification and provides a precise readout of total counts of a canonical miRNA sequence and its two abundant isomiRs present in a sample (**Table S1**). We found that miRNAs with increased addition following enzyme suppression showed a significant decrease in total abundance compared to miRNAs with unchanged (p-value <0.001, t-test) or decreased 3′ addition (p-value = 0.002, t-test) (**Figure 1B**). These results support a model in which the acquisition of increased 3′ additions may serve as a signal for miRNA decay in humans. Our findings complement recent observations of increased addition to an exogenously expressed miRNA during its decay in humans cells [34], in addition to work from plants and algae revealing strong associations between nucleotide additions and miRNA degradation [29]–[31]. Importantly, although increased addition was correlated with decreased miRNA levels, we did not find miRNAs with decreased addition to show increased abundance (**Figure 1B**). Until the mechanism of miRNA degradation is understood, it is difficult to know why decreased 3′ addition does not correlate with changes in miRNA levels. One potential possibility is that the acquisition of additional nucleotides by only specific enzymes may serve as a trigger for the degradation process. Because this analysis represented aggregate data from suppression of a panel of various different nucleotidyl transferase enzymes, we next sought to determine whether knockdown of specific enzymes is associated with miRNA abundance changes (**Figure S1B**). For three enzymes–PAPOLG, ZCCHC11, and TUT1–we again found that increased 3′ addition was associated with decreased miRNA abundance (**Figure 1C**). For two other enzymes, PAPD5 and ZCCHC6, we did not see changes in the abundance of miRNAs that displayed increased 3′ addition (**Figure S1B**), indicating that this phenomenon may occur in an enzyme-specific manner.

Because changes in 3′ additions can involve the loss or gain of a diverse panel of nucleotides, we next investigated whether certain nucleotides are associated with characteristic changes in abundance. We classified each miRNA isomiR into an addition category based on the first nucleotide of the 3′ addition (e.g., A, AA, and AU comprise the “A” family), filtered for categories that included a minimum of at least 5 miRNAs, and compared the abundance changes associated with significant increase or decrease of each class of miRNA additions (**Figure 1D**). We found substantial decreases in the total expression of miRNAs featuring increased 3′ U addition (p-value <1×10^−5^, t-test), with most miRNAs showing a 50% reduction compared to miRNAs with unchanged additions. Together, these results reveal a strong association between miRNA 3′ nucleotide modifications and abundance levels. While future *in vivo* and *in vitro* assays for miRNA turnover will be needed to directly address the differential stability of miRNA isomiRs, these experiments indicate that the acquisition of a 3′ U family addition is associated with decreased miRNA expression.

### The Nucleotidyl Transferase Enzyme TUT1 Broadly Regulates miRNA Abundance

**Figure 2:**
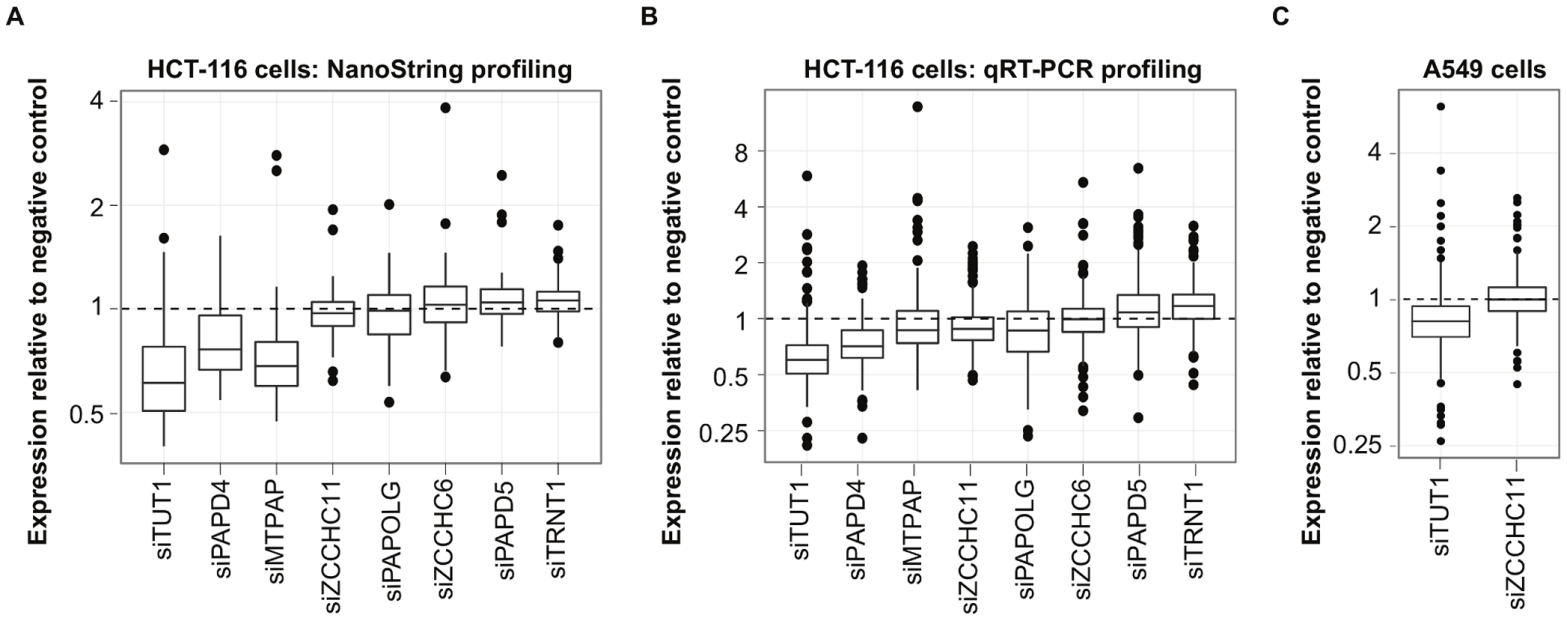
TUT1 suppression yields broad decreases in miRNA expression. We performed miRNA profiling to determine the effect of suppressing nucleotidyl transferase enzymes in HCT-116 cells on miRNA abundance. (**A**) We compared the abundance of miRNAs detectable with at least 50 counts on the NanoString platform in the enzyme suppressed vs. negative control siRNA-transfected cells. Boxplots depict miRNA expression relative to negative control cells, which are depicted by the horizontal line at 1. Three enzymes–TUT1, PAPD4, and MTPAP–showed a significant, broad decrease in miRNA abundance (t-test, p-value <1×10^−10^). (**B**) Boxplots depict the average miRNA expression values from qRT-PCR profiling of over 300 miRNAs in the enzyme-suppressed and negative control cells. In this independent sample set, we found that suppression of TUT1 and PAPD4 lead to highly significant decreases in the abundance of the vast majority of miRNAs analyzed (t-test p-value <1×10^−30^ for both enzymes). (**C**) Suppression of TUT1 in A549 lung carcinoma cells yields decreased miRNA abundance. qRT-PCR arrays were used to compare the global miRNA expression levels in A549 cells transfected with siRNAs against TUT1 or a control nucleotidyl transferase, ZCCHC11, versus negative-control treated cells. A549 cells showed a significant reduction in miRNA abundance following TUT1 suppression (t-test, p-value <1×10^−20^), while ZCCHC11 suppression did not affect miRNA levels.

While examining the enzyme-specific changes in miRNA abundance levels, we noticed that suppression of one nucleotidyl transferase enzyme, TUT1, resulted in striking decreases in miRNA abundance. Remarkably, miRNAs with unchanged, increased, and decreased 3′ addition all showed a substantial reduction in expression in cells with TUT1 knockdown compared to cells transfected with a negative control siRNA (**Figure 1C, right panel**), suggesting this phenomenon could occur through a mechanism independent of nucleotide additions by TUT1. To determine whether the TUT1 enzyme and potentially other nucleotidyl transferase enzymes affect miRNA abundance more broadly, we examined the expression of each of the 65 miRNAs robustly detected with the NanoString platform, regardless of whether the miRNA showed changes in additions. In this set of miRNAs, we found that whereas suppression of most of the nucleotidyl transferases had minimal effects on miRNA abundance, knockdown of TUT1 produced substantial decreases in overall miRNA abundance, as did suppression of PAPD4 and MTPAP (**Figure 2A** **,** t-test adjusted p-value <1×10^−10^ for all three enzymes). The greatest magnitude of reduction in miRNA abundance was seen following TUT1 suppression, which resulted in a median relative miRNA expression value of 0.61 compared to negative control cells (**Table 1**). These results suggest that while the majority of nucleotidyl transferases do not broadly affect miRNA levels, the TUT1 enzyme is required for maintaining miRNA expression in human cells.

**Table 1:** Suppression of nucleotidyl transferases affects overall miRNA abundance.

	NANOSTRING		qRT-PCR	
Enzyme	Mean RQ	Median RQ	Mean RQ	Median RQ
MTPAP	0.76	0.68	1.04	0.85
PAPD4	0.81	0.76	0.76	0.71
PAPD5	1.08	1.04	1.20	1.07
PAPOLG	1.00	0.99	0.90	0.84
TRNT1	1.06	1.06	1.20	1.16
TUT1	**0.69**	**0.61**	**0.69**	**0.60**
ZCCHC11	0.99	0.97	0.94	0.87
ZCCHC6	1.09	1.03	1.06	0.99

We profiled miRNA expression with the NanoString nCounter platform (left) and miRNA qRT-PCR assays (right) following suppression of a panel of nucleotidyl transferases. Suppression of TUT1, shown in bold, led to the most substantial decreases in miRNA expression compared to cells treated with negative control siRNAs (t-test adjusted p-value <1×10^−10^ for both NanoString and qRT-PCR platforms). The table depicts the mean and median relative expression values (RQ) of miRNAs robustly detected across all samples. This included 65 miRNAs from NanoString profiling and 217 miRNAs from qRT-PCR arrays.

To confirm and extend these results more broadly, we used Exiqon qRT-PCR arrays to globally profile the expression of over 300 miRNAs in HCT-116 cells following the suppression of our panel of eight nucleotidyl transferase enzymes. Exiqon miRNA qRT-PCR assays are based on a 3′ tailing reaction, and therefore allowed us to detect the total abundance of both canonical and isomiR miRNAs in a single reaction. In an independent series of transfections, we again found that suppression of the majority of the enzymes had very minimal effects on miRNA abundance (**Figure 2B**). In contrast, when we suppressed TUT1, we again found substantial decreases in the vast majority of miRNAs **(** **Figure 2B** **)**, which featured a median relative expression value of 0.60 in the siTUT1 cells compared to cells treated with negative control siRNAs (t-test, adjusted p value <1×10^−40^) (**Table 1**). Suppression of PAPD4 also led to a highly significant reduction in miRNA expression, (t-test adjusted p value <1×10^−30^), although the median relative miRNA expression value of 0.71 in siPAPD4 cells was of a smaller magnitude than observed from TUT1 suppression. As an additional control, we performed Western blots for the TUT1 protein and found that RNAi effectively reduced expression of TUT1 at the protein level (**Figure S1C**). Together, these results indicate that the loss of TUT1 has broad effects on miRNA expression levels that extend beyond its measurable effects on 3′ additions.

We next tested whether this phenomenon extended to other cell lines. In A549 lung carcinoma cells, we suppressed TUT1 and used Exiqon miRNA qRT-PCR assays to quantify global changes in miRNA abundance. We again found that suppression of TUT1 resulted in significant decreases in miRNA expression levels (**Figure 2C**, t-test p-value <1×10^−20^). This effect was specific to TUT1 knockdown, as we did not observe a change in miRNA abundance following suppression of another nucleotidyl transferase serving as a negative control (i.e., ZCCHC11).

### TUT1 as a Novel Regulator of miRNA Expression

**Figure 3:**
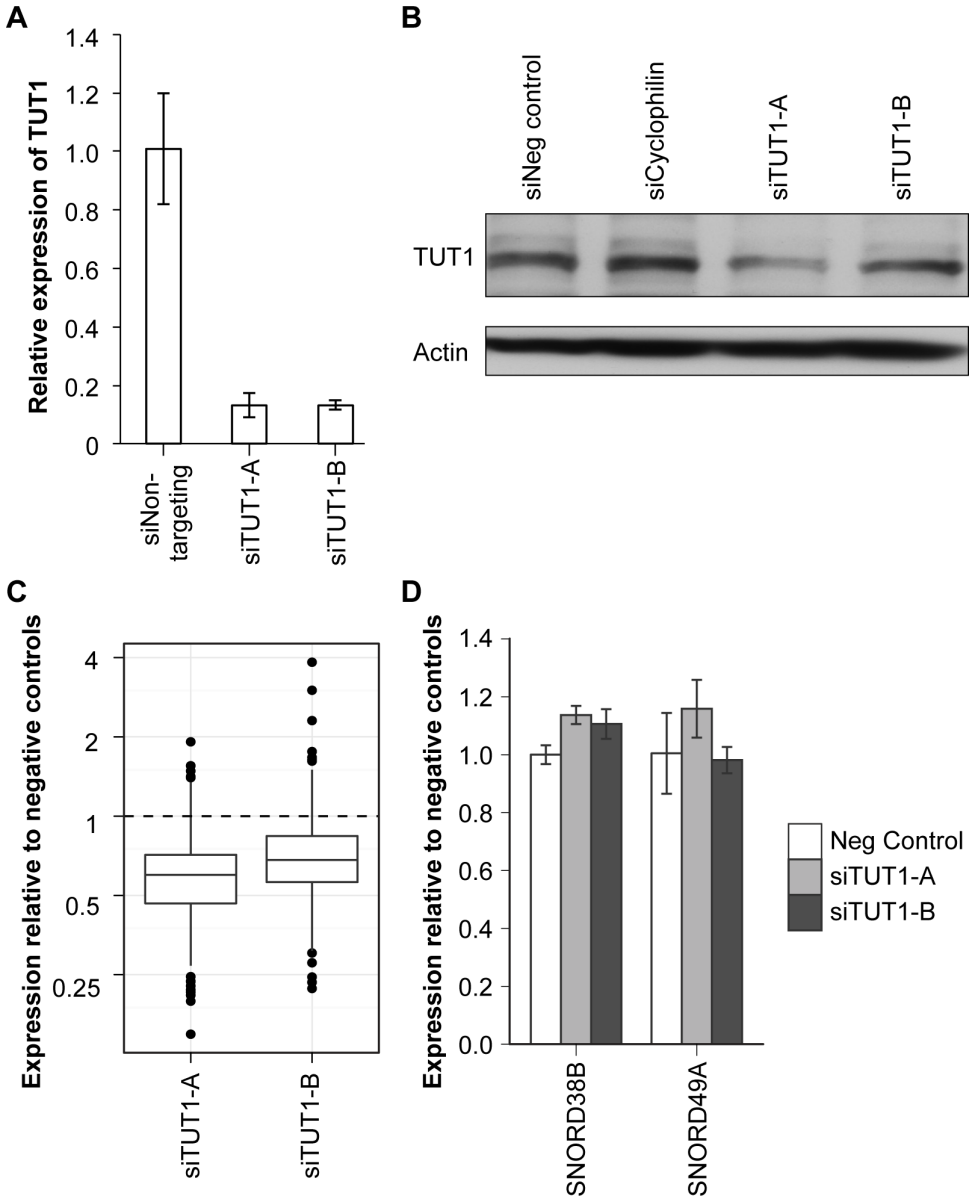
TUT1 knockdown with additional, independent siRNAs confirms the role of TUT1 in maintaining miRNA expression levels. (**A**) Two additional TUT1 siRNAs were used to suppress TUT1 in HCT-116 cells. Bar graphs show qRT-PCR assessement of the suppression of TUT1 compared to cells treated with negative control siRNAs. (**B**) RNAi with both siTUT1-A and siTUT1-B decreased the expression of the TUT1 protein in HCT-116 cells. (**C**) Exiqon miRNA qRT-PCR arrays were used to compare the global miRNA expression in enzyme suppressed versus negative control cells. Boxplots depict miRNA expression following TUT1 suppression with each of the two new TUT1 siRNAs. Points below the dashed line indicate decreased miRNA abundance with TUT1 suppression compared to the negative control cells. (**D**) Bar graphs compare the expression of two small nucleolar RNAs, SNORD38B and SNORD49A, in TUT1 supppressed versus control cells. TUT1 suppression did not affect small nucleolar RNA abundance levels, which confirms the specificity of the phenotype to miRNAs.

To verify that the reductions in miRNA abundance seen upon TUT1 suppression were not due to off-target effects of our siRNAs, we repeated these experiments with two additional TUT1 siRNAs that have previously been shown to potently suppress the TUT1 protein [36]. We confirmed that RNAi decreased both the levels of the TUT1 transcript (**Figure 3A**) and the TUT1 protein (**Figure 3B**). Next, when we examined the resulting changes in miRNA abundance, we again found that suppression of TUT1 led to decreased miRNA expression compared to cells treated with negative control siRNAs, with a median expression value of 0.60 and 0.68 for the two different siRNAs, with the siRNA that more potently suppressed the TUT1 protein (siTUT1-A) displaying slightly greater effects on miRNA abundance (**Figure 3C** **,** t-test p-value <1×10^−30^ for both TUT1 siRNAs). To determine whether this effect is specific to miRNAs, we also examined the expression of two small nucleolar RNAs assayed in parallel on the qRT-PCR miRNA array. We did not find significant changes in the expression of SNORD38B or SNORD49A following TUT1 suppression **(** **Figure 3D**), which indicates that the decrease in miRNAs seen upon TUT1 suppression is unique to this particular class of small RNAs.

### Potential Mechanisms of TUT1-mediated Effects on miRNA Abundance

TUT1 could potentially affect miRNA levels via indirect or direct mechanisms. TUT1 has previously been described to act in the nucleus as a poly (A) and (U) polymerase for a variety of substrates [36], [37]. We thus hypothesized that TUT1 could potentially affect miRNA primary transcript abundance. Using qRT-PCR arrays to measure the expression of a panel of over 70 primary transcripts, we found that TUT1 suppression did not lead to decreased expression of primary transcripts (**Figure S2A**), rather there was a slight increase in the primary transcripts for most miRNAs, suggesting that TUT1 affects subsequent steps in miRNA processing and/or turnover. We considered whether TUT1 could indirectly regulate miRNAs by affecting the mRNA expression of miRNA processing enzymes, as TUT1 was previously found to regulate the stability of a subset of mRNA transcripts [36]. However, we found no change in the expression of *Dicer*, *Drosha*, or the Drosha co-factor *DGCR8* following TUT1 suppression (**Figure S2B**). Thus, while the precise mechanism by which TUT1 maintains miRNA levels remains unknown, our results suggest that TUT1 acts at a post-transcriptional step. Recent work has shown that uridylation of specific precursor miRNAs by the ZCCHC11 enzyme can prevent miRNA maturation [40], [41]. Future studies to determine whether TUT1 can affect the abundance or 3′ additions of precursor miRNAs should reveal whether the activity of TUT1 is exclusively restricted to mature miRNAs.

Our work investigates the functional effects of miRNA 3′ additions by nucleotidyl transferases on a global scale. We found that the acquisition of 3′ additions–particularly of a 3′ U–is associated with decreased miRNA abundance. We also determined that the TUT1 nucleotidyl transferase globally regulates miRNA abundance and represents a newly identified post-transcriptional regulator of miRNA homeostasis in the cell. Future work will be needed to determine the mechanism by which TUT1 regulates miRNA abundance. Our results indicate that this is an indirect mechanism independent of regulation of miRNA 3′ NTA by TUT1, based on our observation that knockdown of TUT1 affected miRNA abundance broadly while only leading to changes in 3′ additions of a subset of the miRNAs we examined. However, we cannot exclude the possibility that TUT1 effects occur predominantly through direct nucleotide additions that we did not detect because they lead to rapid miRNA degradation. Because TUT1 suppression led to increased 3′ U addition on many miRNAs, our results support a model in which uridylation may serve as a signal for rapid miRNA decay in human miRNAs. This work extends a recent report that found an increase in 3′ uridylation during the decay of an exogenously expressed miRNA [34], to include a potential mechanism of degradation for endogenous human miRNAs. Taken together, our studies indicate that TUT1 provides an important layer of regulation for miRNA abundance, and serve as an entry point for future work to fully understand the mechanisms that regulate miRNA accumulation.

## Materials and Methods

### Cell Culture and Transfections

HCT-116 cells were obtained from Bert Vogelstein and maintained in McCoy’s media (Invitrogen) with 10% FBS (Atlanta Biologicals). A549 cells were obtained from ATTC and maintained in DMEM (Invitrogen) with 10% FBS. Transfections with biological replicates were performed in 6-well dishes with 62,500 cells transfected with 66 nM siRNA using Lipofectamine RNAiMax (Invitrogen) according to the manufacturer’s instructions. Dharmacon On-Target+ SMARTpool siRNAs were used for suppression of a panel of eight nucleotidyl transferases as previously described [25]. SMARTpool siRNAs against the housekeeping gene *Cyclophilin B* was used as the negative control (Dharmacon). TUT1 siRNAs described by Mellman et al. [36] were synthesized with an unmodified backbone (IDT). Non-targeting siRNAs with an unmodified backbone that do not show homology to any known mammalian gene were used as a negative control for these siRNAs (siAll-Stars, Qiagen).

### RNA Isolation and qRT-PCR

72 hours post transfection with siRNAs, cells were washed with PBS and lysed in 700 uL Qiazol (Invitrogen). RNA was isolated using the miRNeasy RNA isolation kit (Qiagen), quantified with a spectrophotometer (Bio-Tek), and normalized to equal concentration. Taqman assays from Applied Bioystems were used to measure the degree of nucleotidyl transferase suppression. The expression of the endogenous control genes *GUSB* and *GAPDH* were used for normalization. For primary miRNA qRT-PCR profiling, Taqman assays were obtained from Applied Biosystems for 73 different primary miRNAs (assay numbers listed in **Table S2**). Primary miRNAs were assayed with two technical replicates according to the manufacturer’s instructions. For primary miRNA data analysis, we removed any miRNA with an undetermined cycle threshold (C_T_) or with C_T_ >35 in any sample, which yielded 48 pri-miRNAs for subsequent analysis. Delta C_T_ values were calculated using the average of three endogenous control genes run on each plate: GAPDH-FAM, ACTB-FAM, and GAPDH-VIC. Delta delta C_T_ values to the siCyclophilin negative control cells were calculated, and relative expression values were determined.

Exiqon V2.0 Panel 1 Human miRNA qRT-PCR arrays in 384-well format were used to assess global changes in miRNA levels. Reverse transcription was performed using the Universal cDNA Synthesis kit (Exiqon) using equal amounts of RNA within each sample set. Real-time PCR was performed using a Viia7 instrument (Applied Biosystems), and all the data were imported into a single gene expression study and thresholded to 0.2 across all arrays to minimize plate to plate variation. Our approach to normalization was to first evaluate whether using the two endogenous normalizer control small RNAs (i.e., snoRNAs SNORD38b and SNORD49a) assayed on each qRT-PCR array led to a reduction in the standard deviation between biological replicates. If technical variation that can be normalized using endogenous controls is a significant component of the observed variation between samples, then we would expect endogenous control normalization to reduce the standard deviation between biological replicates. In the case of the HCT-116 cell experiments, endogenous control normalization did not decrease the variation in biological replicates, suggesting that technical variation was minimal compared to meaningful biological variation. Thus, for HCT-116 we have presented miRNA data not normalized to any additional RNAs. In the A549 cell experiments, we found that biological replicates showed decreased variation following snoRNA normalization, which suggested this normalization was an appropriate correction for technical variation (e.g., variations in RNA input). Therefore, we have presented the normalized data for experiments done with this cell line. Next, C_T_ values were filtered to maintain only reliably detected miRNAs (requiring C_T_ <35 across all samples). Delta C_T_ values were then calculated relative to cells treated with negative control siRNAs, and relative expression values were computed. Two-sided t-tests were used to compare the miRNA expression in enzyme suppressed versus control cells. The multtest package of R from Bioconductor [42] was used to calculate adjusted p-values using the Benjamini-Hochberg method.

### Western Blots

For protein quantification, HCT-116 cells were lysed in RIPA buffer 72 hours post transfection with siRNAs. Protein samples were resolved on Tris-Glycine gels (Invitrogen), and Western blots were performed using TUT1 antibody from Sigma (SAB2102610) on nitrocellulose membranes blocked with 5% non-fat milk.

### NanoString nCounter Data Analysis

For NanoString quantification of 3′ additions, we performed additional analyses on data collected from previously described samples [25]. MicroRNAs were filtered to include only those expressed with at least 50 counts (the total of both canonical and isomiR sequences) for our NanoString abundance analyses. Two-sided t-tests were used to assess the abundance changes associated with 3′ addition changes.

The NanoString data from this study are available at the NCBI Gene Expression Omnibus (http://www.ncbi.nlm.nih.gov/geo) under accession no. GSE26970.

## Supporting Information

Figure S1 **Nucleotidyl transferase suppression in HCT-116 cells.** **(A)** qRT-PCR was used to evaluate potential compensatory changes in nucleotidyl transferase expression levels following knockdown of the TUT1 enzyme. **(B)** Boxplots display the changes in miRNA abundance following the suppression of each enzyme. MicroRNAs are categorized by whether they showed unchanged, increased, or decreased 3′ additions. MicroRNA abundance was significantly decreased in miRNAs with increased additions compared to unchanged additions following the suppression of PAPOLG and ZCCHC11 (t-test p-value <0.05). (**C**) Western blot confirms the suppression of TUT1 following treatment of HCT-116 cells with TUT1 siRNAs from Dharmacon. Both lanes are from a single Western blot, with non-relevant lanes omitted and spaces indicating non-adjacent lanes.
(TIF)

Figure S2 **TUT1 does not regulate the** **mRNA expression of miRNA primary transcripts or miRNA processing enzymes.** (**A**) The expression of 73 primary miRNA transcripts was measured with qRT-PCR following TUT1 suppression in HCT-116 cells. Scatterplots depict the delta CT values in the enzyme suppressed versus control cells. Suppression of TUT1, PAPD4, and ZCCHC11 all yielded slight increases in pri-miRNA abundance, indicating that changes in mature miRNA levels following TUT1 loss are not a result of upstream decreases in primary miRNA transcript abundance. (**B**) qRT-PCR was used to assess the expression of three miRNA processing components following knockdown of a panel of nucleotidyl transferases in HCT-116 cells. Although the expression levels of these genes are slightly variable following nucleotidyl transferase knockdown, suppression of TUT1 does not yield unique changes that account for the global decreases in miRNA abundance.
(TIF)

Table S1 Table of NanoString normalized miRNA counts and filtered miRNAs used in the analyses.
(XLSX)

Table S2 Table of miRNA primary transcript assays from Applied Biosystems.
(XLS)
