# Supplementary figures and images for: The Human TUT1 Nucleotidyl Transferase as a Global Regulator of microRNA Abundance
Source: PLoS One. 2013 Jul 18;8(7):e69630. doi: 10.1371/journal.pone.0069630 (PMC3715485; doi:10.1371/journal.pone.0069630)

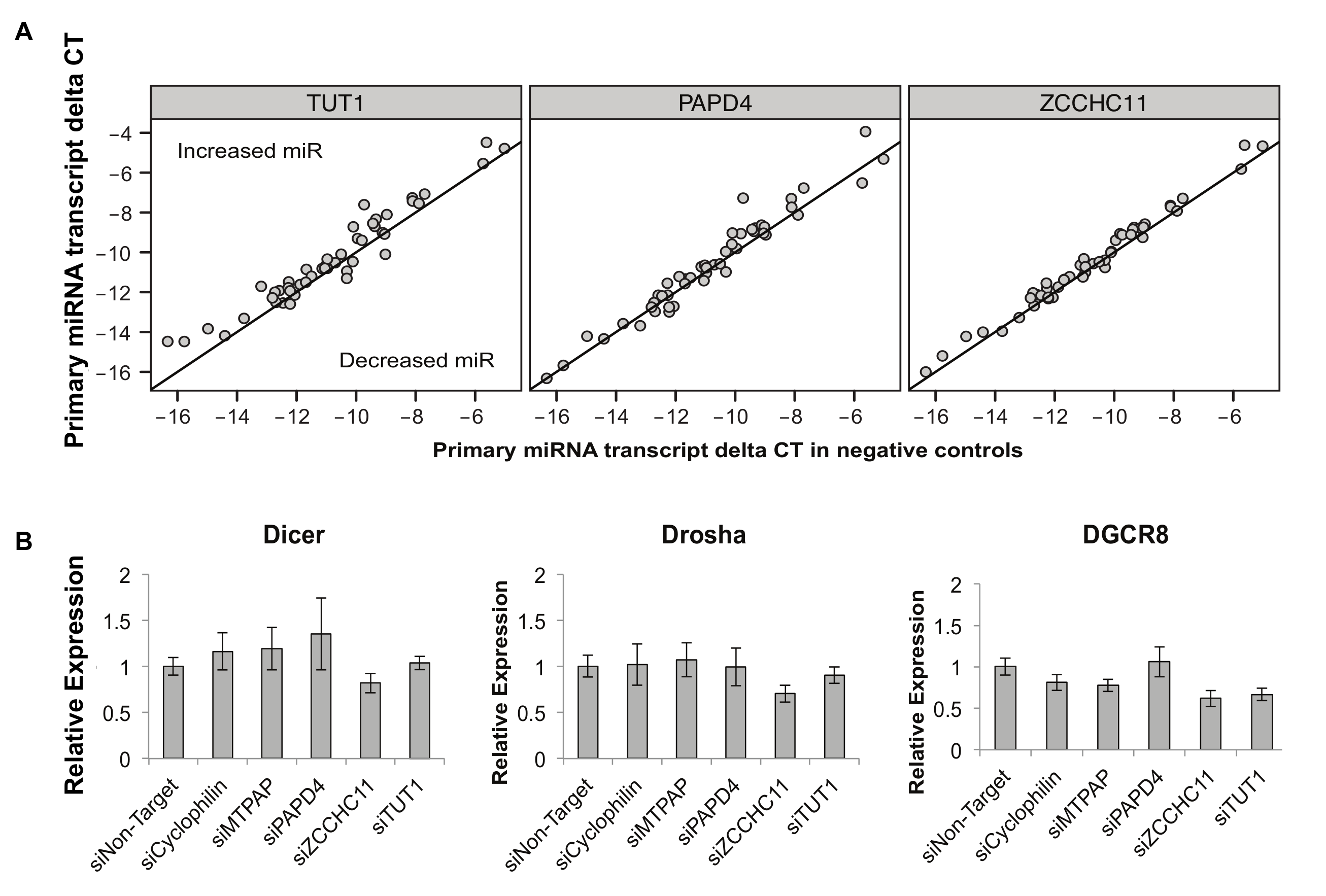

Supplement: Figure S2 — TUT1 does not regulate the mRNA expression of miRNA primary transcripts or miRNA processing enzymes. (A) The expression of 73 primary miRNA transcripts was measured with qRT-PCR following TUT1 suppression in HCT-116 cells. Scatterplots depict the delta CT values in the enzyme suppressed versus control cells. Suppression of TUT1, PAPD4, and ZCCHC11 all yielded slight increases in pri-miRNA abundance, indicating that changes in mature miRNA levels following TUT1 loss are not a result of upstream decreases in primary miRNA transcript abundance. (B) qRT-PCR was used to assess the expression of three miRNA processing components following knockdown of a panel of nucleotidyl transferases in HCT-116 cells. Although the expression levels of these genes are slightly variable following nucleotidyl transferase knockdown, suppression of TUT1 does not yield unique changes that account for the global decreases in miRNA abundance. (TIF) [file pone.0069630.s002.tif]
